# The life cycle of a capillary: Mechanisms of angiogenesis and rarefaction in microvascular physiology and pathologies

**DOI:** 10.1016/j.vph.2024.107393

**Published:** 2024-06-08

**Authors:** Declan Manning, Ernesto J. Rivera, L. Fernando Santana

**Affiliations:** Department of Physiology & Membrane Biology, School of Medicine, University of California, Davis, United States of America

**Keywords:** Angiogenesis, Arteriogenesis, Microvascular rarefaction, Hypertension, Vascular dementia, Heart failure with preserved ejection fraction, Pericytes, Small vessel disease

## Abstract

Capillaries are the smallest blood vessels (<10 μm in diameter) in the body and their wallsre lined by endothelial cells. These microvessels play a crucial role in nutrient and gas exchange between blood and tissues. Capillary endothelial cells also produce vasoactive molecules and initiate the electrical signals that underlie functional hyperemia and neurovascular coupling. Accordingly, capillary function and density are critical for all cell types to match blood flow to cellular activity. This begins with the process of angiogenesis, when new capillary blood vessels emerge from pre-existing vessels, and ends with rarefaction, the loss of these microvascular structures. This review explores the mechanisms behind these processes, emphasizing their roles in various microvascular diseases and their impact on surrounding cells in health and disease. We discuss recent work on the mechanisms controlling endothelial cell proliferation, migration, and tube formation that underlie angiogenesis under physiological and pathological conditions. The mechanisms underlying functional and anatomical rarefaction and the role of pericytes in this process are also discussed. Based on this work, a model is proposed in which the balance of angiogenic and rarefaction signaling pathways in a particular tissue match microvascular density to the metabolic demands of the surrounding cells. This negative feedback loop becomes disrupted during microvascular rarefaction: angiogenic mechanisms are blunted, reactive oxygen species accumulate, capillary function declines and eventually, capillaries disappear. This, we propose, forms the foundation of the reciprocal relationship between vascular density, blood flow, and metabolic needs and functionality of nearby cells.

## 1. Introduction

The cardiovascular system is composed of the heart, which functions like a pump, and arteries, arterioles, capillaries, venules, and veins, that create a network of pipe-like structures that transport blood to and from all organs of the body during each cardiac cycle. Arteries and arterioles share a common anatomical design. They are composed of an inner mono layer of endothelial cells which is wrapped by smooth muscle cells (SMCs) running perpendicular to the longitudinal axis of these vessels. By contrast, capillaries are composed of endothelial cells and specialized auxiliary cells called pericytes. The basal lamina, also known as the basement membrane —a thin, fibrous, extracellular matrix— provides structural support to arteries, arterioles, and capillaries.

The canonical view of capillaries is that they are the sites where gas exchange (i.e., O_2_ and CO_2_) as well as transport of nutrients and hormones to tissues throughout the body takes place. Capillary endothelial cells control this by regulating cell-to-cell connectivity and hence the diffusion of substances, either between cells or through intracellular mechanisms (e.g., transcytosis).

Seminal work from the Nelson lab [1–3] identified a novel function of capillary endothelial cells: electrical signaling hubs that trigger changes in upstream arteriolar diameter via changes in smooth muscle contractility to match blood flow to local metabolic demands (i.e., functional hyperemia). In the brain, this is known as neuro-vascular coupling.

Angiogenesis is the physiological process through which new blood vessels form from pre-existing vessels, playing a crucial role in both healthy and pathological conditions such as wound healing, cancer growth, and the development of collateral circulation following ischemia. Conversely, microvascular rarefaction refers to the loss capillaries within a tissue, leading to reduced blood flow and oxygen supply. This process is commonly associated with diseases such as hypertension, diabetes, and chronic heart disease, resulting in tissue hypoxia and dysfunction. In this review, we explore the cellular and molecular mechanisms driving angiogenesis and rarefaction, highlighting their functional impact on capillary transport and electrical signaling in health and disease.

## 2. How does a blood vessel grow?

During development, de novo blood vessel formation builds a network of blood vessels to supply blood to organs and tissues throughout the developing embryo. This process involves the differentiation of hemangioblast cells, which develop into the preliminary vascular system in a complex developmental process called “vasculogenesis”. Readers interested in learning more about vasculogenesis are encouraged to read a recent review by Potente and Mäkinen [4]. Herein, we will discuss the general mechanisms by which new blood vessels are grown in adulthood.

**Fig. 1.**
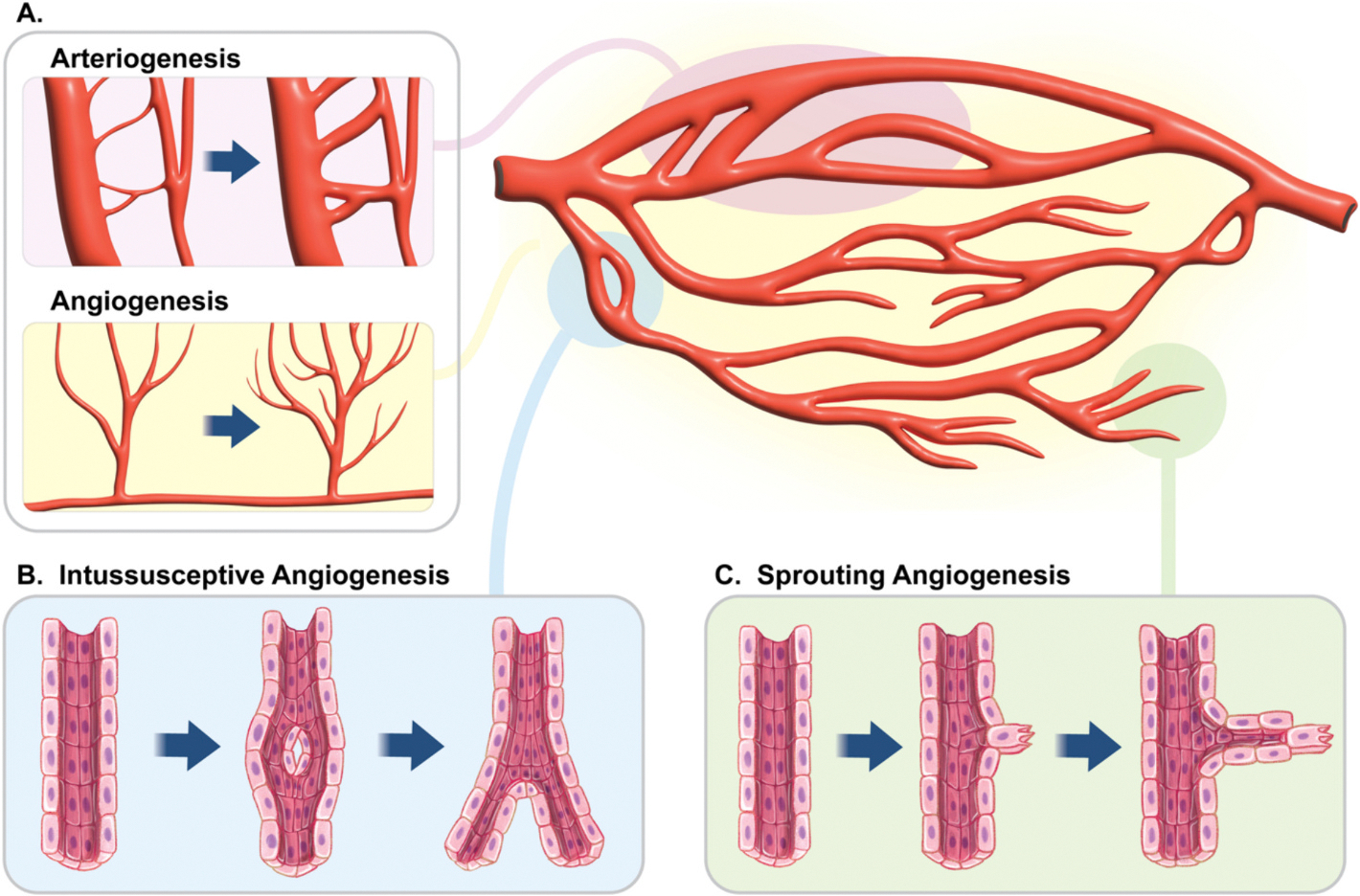
How a blood vessel is born. Cartoons depicting arteriogenesis and angiogenesis (A) as well as intussusceptive (B) and sprouting (C) angiogenesis.

After embryonic development new blood vessels are born and existing vessels adapt to changes in tissue metabolic requirements and blood flow. In general terms, the mechanisms leading to the creation and structural expansion of new vessels are referred collectively as angiogenesis and arteriogenesis (Fig. 1) [5]. We discuss these models here.

### 2.1. Arteriogenesis

Arteriogenesis refers to the structural remodeling of arterioles and arteries through an increase in the caliber of pre-existing vessels [5] (Fig. 1A). This process is distinct from angiogenesis and involves the growth and proliferation of SMCs and endothelial cells in response to elevated intralumenal shear stress [6]. The result is an increase in the diameter of arteries and arterioles that decreases resistance, increasing blood flow to ischemic tissues [7].

**Fig. 2.**
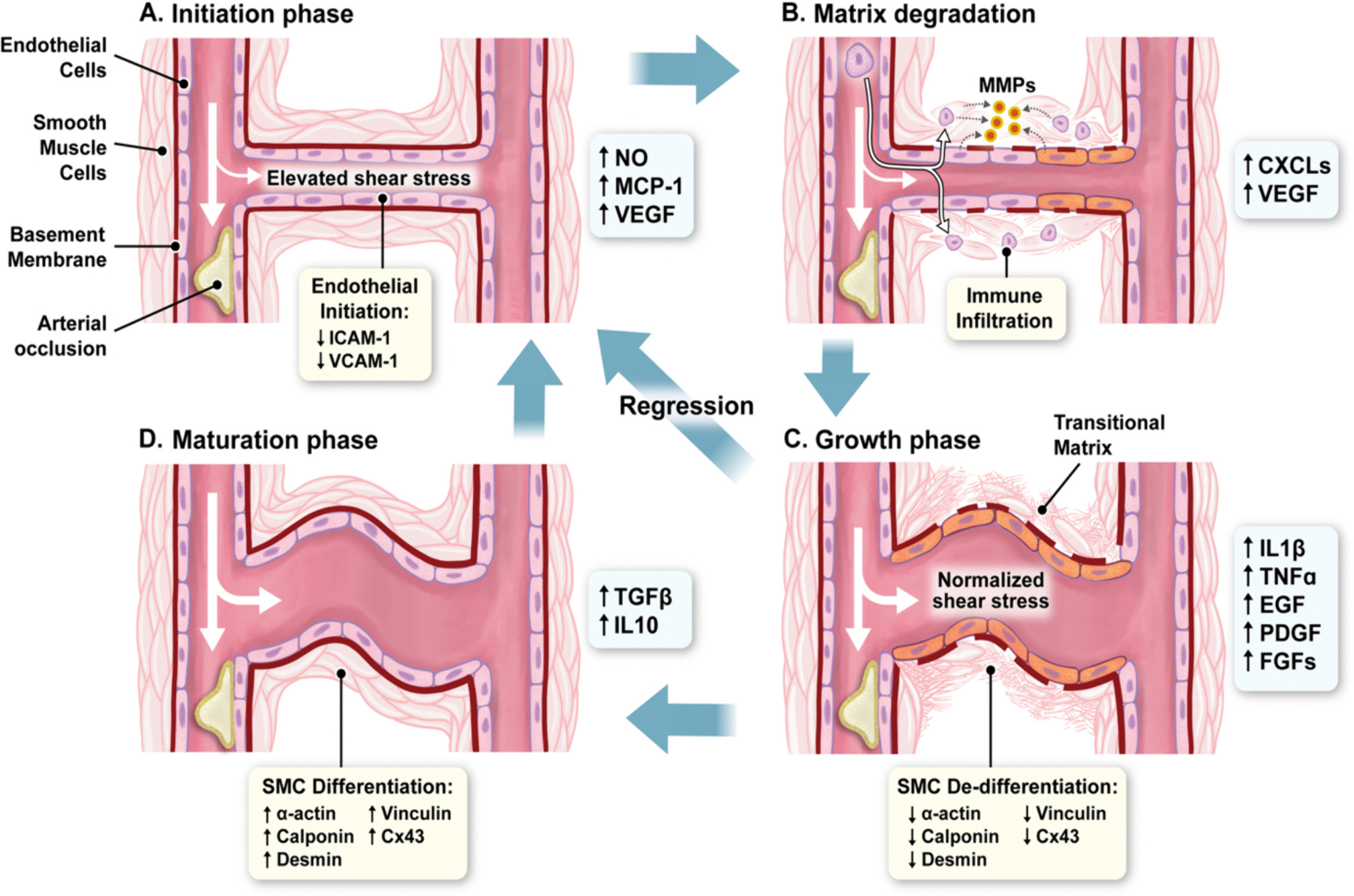
The mechanism of arteriogenesis. The sequence of events underlying arteriogenesis (initiation (A), matrix degradation (B), growth (C), and maturation (D)), illustrated in cartoon form.

Arteriogenesis is triggered by elevated blood turbulence and shear stress across pre-formed anastomoses between different arterial territories [6]. Endothelial shear stress and changes in blood viscosity and flow velocity the most important trigger for arteriogenesis, at least in the heart [6]. These elevations in shear stress induce the production of nitric oxide (NO), vascular endothelial growth factor (VEGF), and monocyte chemoattractant protein-1 with consequent activation, proliferation, and chemotaxis of monocytes (Fig. 2A) [8,9]. VEGF activates vascular and immune cells to release matrix metalloproteinases (MMPs), which degrade the vessels extracellular matrix (Fig. 2B) [10]. This allows for the structural expansion of the vessel due to smooth muscle growth and proliferation (Fig. 2C), culminating in increased diameter and length of the pre-existing anastomotic collaterals (Fig. 2D) [7].

### 2.2. Angiogenesis

The chain of events that initiate angiogenesis start with the development of hypoxia (i.e., low oxygen levels), which stimulates the production of growth factors like VEGF in multiple cells [5,11]. In this section, we discuss two types of angiogenic modalities: intussusceptive angiogenesis and sprouting angiogenesis. These will be discussed here in a physiological context; for an illustration of how these mechanisms are exploited in cancer, see a recent review by Wälchli et al. [12].

During intussusceptive angiogenesis, vessels split to form two distinct capillaries (Fig. 1B) [13]. Like arteriogenesis, this process can be triggered by conditions of high intralumenal shear stress and NO signaling [14]. However, intussusceptive angiogenesis is also driven by hypoxia and thus enhanced VEGF production, often occurring alongside, or secondary, to the canonical angiogenic mechanism: sprouting angiogenesis [14].

**Fig. 3.**
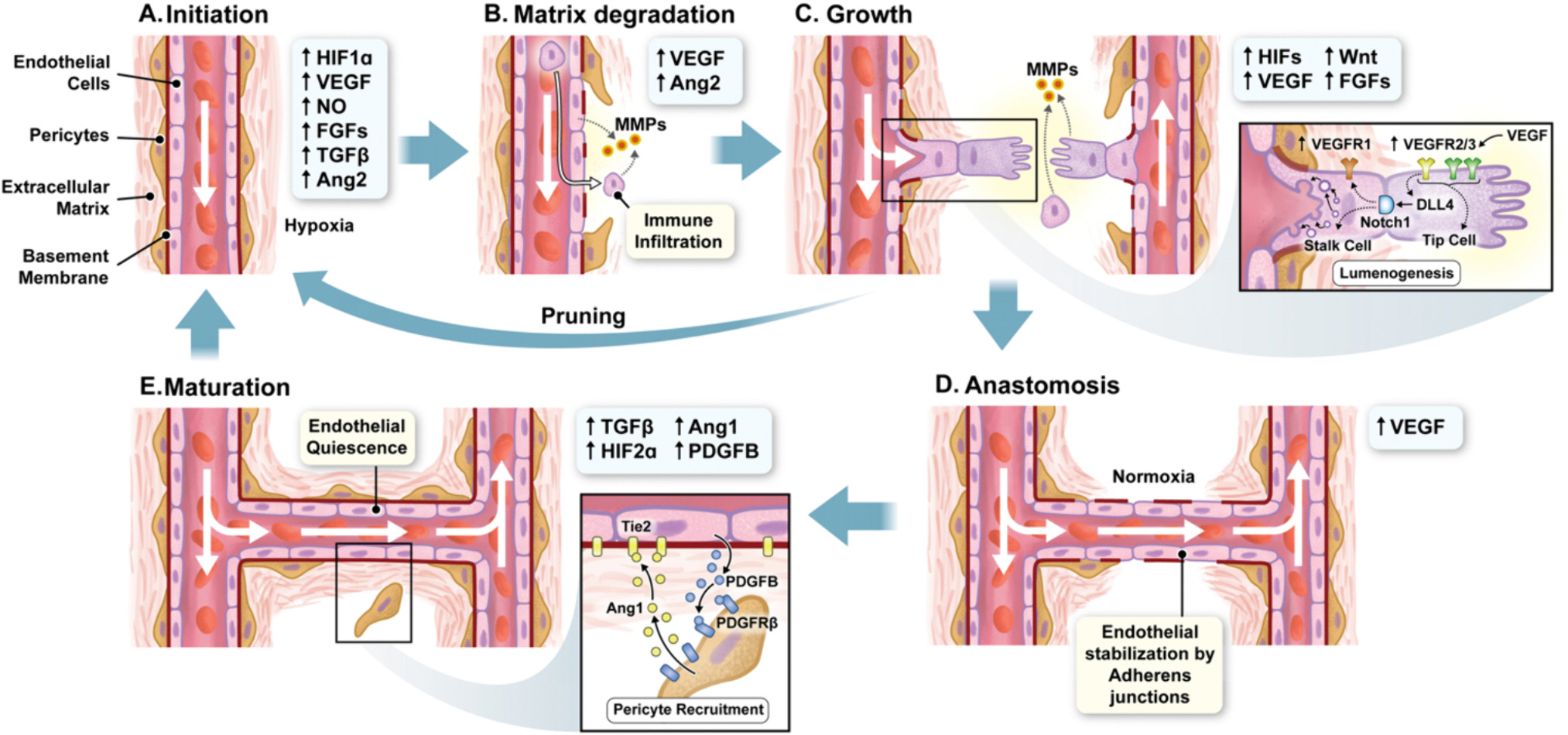
The mechanisms of angiogenesis. The sequence of events underlying angiogenesis (initiation (A), matrix degradation (B), growth (C), anastomosis (D), and maturation (E)), illustrated in cartoon form.

Sprouting angiogenesis is probably the best understood angiogenic modality (Fig. 1C) [5,11]. It starts when increased levels of VEGF induce vasodilation and heightened permeability of nearby blood vessels, facilitating the influx of immune cells and growth factors into the target tissue (Fig. 3A) [15,16]. The binding of VEGF to its receptors on endothelial cells upregulates the expression of proteolytic MMPs, which degrade the basement membrane (Fig. 3B) [17]. This is accompanied by extensive immune cell recruitment, some of which assist in degrading the basement membrane, whilst others secrete Wnt molecules, stimulating a range of endothelial receptors to drive their differentiation [18]. A local accumulation of plasminogen activators and hypoxia-induced hypoxia-inducible factor (HIF) signaling further enforce MMP expression, activation, and function, and so further break down the basement membrane, and inter-endothelial connections whilst disrupting connections with pericytes [19].

Endothelial VEGFR2 signaling disrupts endothelial cell-cell junctions through focal adhesion kinase (FAK) signaling [20] as well as Orai1 and TRP channel Ca^2+^ entry [21]. These Ca^2+^ signals also drive endothelial cells to contract, pulling apart the disrupted cell-cell junctions and exposing the surrounding tissue to angiogenic growth factors and immune cells. Overall, this process cultivates a fertile environment for capillary growth. On the other hand, VEGF-induced endothelial permeability in arteriogenesis has not been examined in-depth and questions remain about the distinctions underlying arterial and capillary ECM reconstruction in arteriogenesis and angiogenesis, respectively.

Endothelial cells respond to VEGF in divergent ways during the growth stage of sprouting angiogenesis (Fig. 3C) [22]. When VEGF activates VEGFR 2 and 3, a broad intracellular signal cascade hastens glycolytic processes and shifts a cell into the ‘tip’ phenotype [22]. The tip cell is highly mobile, migrating towards the source of VEGF whilst secreting proteolytic enzymes from its invasive filopodia [22,23]. Crucially, tip cell VEGFR2 signaling also activates intracellular delta-like 4 protein (DLL4) which traffics to the cell surface where it activates neurogenic locus notch homolog protein 1 (NOTCH1) on neighboring endothelial cells [24]. Briefly, membrane NOTCH1 becomes cleaved, and its intracellular domain (NICD) translocates to the nucleus, where it activates the centromere binding factor-1 (cbf1) transcriptional factor [25]. The transcriptional switch initiated by cbf1 is a key driver towards the distinct ‘stalk cell’ phenotype [25], although NOTCH1 exerts a diverse effect on these cells by also modulating intracellular NFκB, mTORC2, AKT, and Wnt signaling [24].

Jakobsson et al. [23] suggested that stalk cells develop a phenotype defined by enhanced, highly coordinated, proliferation (Fig. 3C). NOTCH1 drives ‘lateral inhibition’, the process coordinating a single tip cell to lead a group of stalk cells [23]. NOTCH1 downregulates expression of VEGFR2 and 3, stifling the signal that might otherwise trigger stalk cells to adopt the tip phenotype [23,26]. Nonetheless, VEGF/VEGFR and DLL4/NOTCH1 signaling are constantly balanced by each endothelial cell, such that a tip cell can change to a stalk cell, and vice versa [23]. This ‘angiogenic shuffling’ endows versatility to the angiogenic sprouting mechanism. Guided by the chemoattractant properties of VEGF and regulated by the VEGF/VEGFR and DLL4/NOTCH1 signaling mechanisms outlined above, activated endothelial cells grow into a tubular structure.

Directed endothelial cell multiplication generates a capillary lumen in the wake of the migratory tip cell (lumenogenesis). Diverse mechanisms of angiogenic lumenogenesis have been observed, mostly involving the formation of vesicles and/or vacuoles inside and/or between nascent capillary endothelial cells, which later merge to form the lumen of the growing capillary [4]. In an important update to these models, Gebala et al. [27] found that tip cells extend the trailing apical membrane through a pressure-driven ‘inverse blebbing’ mechanism. This underscores the importance of blood pressure and shear stress in vascular growth, as even in the early stages of angiogenesis, blood flow into the leaky, developing capillary determines its rate of growth [27].

Notably, not all sprouts formed during angiogenesis will mature into functional vessels. A crucial regulatory step known as pruning comes into play, where excessive vessels are selectively removed through apoptosis [28]. This pruning process ensures the development of an efficient vascular network, preventing excessive vascularization.

Once the new vessel has fused with an existing vessel wall, blood flow is established through it (Fig. 3D) [29]. This ensures that newly generated blood vessels can effectively deliver oxygen and nutrients to the surrounding tissue, supporting its growth and function. The orchestration of these mechanisms is finely tuned, maintained by a delicate balance between pro-angiogenic factors like VEGF and anti-angiogenic factors like angiostatin and endostatin [30]. This balance ensures that angiogenesis occurs when necessary, such as during wound healing or tissue growth, while also preventing excessive or aberrant vessel formation.

Finally, angiogenic endothelial cells produce platelet-derived growth factor B (PDGFB), which activates PDGF receptor β on nearby pericytes [31]. Simultaneously, pericytes express Angiopoietin-1, which activates Tie2 on endothelial cells and pericytes [32]. In combination, these signals recruit pericytes to the newly established blood vessel (Fig. 3E). Pericytes regulate endothelial permeability by inhibiting transcytosis and upregulating endothelial cell tight junction expression in neighboring cells [33]. Moreover, contractile processes enable many pericytes to dynamically regulate capillary blood flow (by controlling lumenal diameter and resistance) and permeability (by reversibly opening inter-endothelial junctions) [34]. Hence, the integration of pericytes signifies the maturity of nascent vessel into a fully functional capillary.

In addition to VEGF and HIF, other growth factors have been implicated in angiogenesis, but their actions are complex. For example, the basic fibroblast growth factor (bFGF or FGF-2), stimulates the proliferation of various cell types, including endothelial cells, fibroblasts, and SMCs, and plays a crucial role in promoting endothelial cell migration and the formation of new blood vessels [35].

The transforming Growth Factor-β (TGFβ) plays a more complex role in angiogenesis, acting as both a promoter and inhibitor depending on the context and signaling pathways involved [36]. It can stimulate angiogenesis by promoting the differentiation and migration of endothelial cells and the production of extracellular matrix components [37]. Conversely, TGFβ can also inhibit angiogenesis by inducing endothelial cell apoptosis and inhibiting endothelial cell proliferation [38]. This dual role is mediated through its signaling via the Smad-dependent pathway for its inhibitory effects and the activation of other pathways such as ALK1 and endoglin for its angiogenic effects [38,39]. TGFβ has been shown to recruit pericytes and induce pericyte differentiation into vascular SMCs [37].

### 2.3. What determines vessel density?

Having discussed the critical role of VEGF, HIF, and TGFβ in regulating both the growth and pruning of new vessels, it is essential to understand the factors that influence overall vessel density. This is an important issue, as vascular density seems to reach steady state levels that vary within and between tissues.

Studies on the effect of exercise provide insight into the dynamic regulation of vascular density in muscle. This work indicates that exercise is associated with a surge in endothelial cell proliferation and VEGF production [40]. Furthermore, a significant upregulation of endothelial nitric oxide synthase (eNOS) mRNA is observed [40], suggesting that eNOS contributes to vascular growth in response to augmented blood flow and mechanical stretch. Notably, VEGF mRNA and matrix metalloproteinase-2 (MMP-2) mRNA levels remain constant, implying that the rise in VEGF protein concentration and its angiogenic effects are controlled post-transcriptionally [40]. These outcomes highlight that enhanced blood flow and passive stretch act as effective physiological stimuli for angiogenesis, shedding light on skeletal muscle’s adaptation to increased vascular demands through VEGF production.

Multiple studies have suggested that the rate of angiogenesis varies between different tissues [41–43]. These varied rates of angiogenesis can be explained by the different metabolic activities, vascular densities, and rates of cellular turnover in each tissue, as evidenced by the high angiogenic activity in the heart, liver, and skin, respectively [41,43]. A detailed investigation of endothelial cell heterogeneity by Marcu et al. [43] confirmed that angiogenic activity in these tissues are determined in part by the expression levels of angiogenic factors (e.g., VEGF) and also by their intrinsic responses to circulating angiogenic factors (e.g., via VEGFR expression). Tissues differentially upregulate these factors following angiogenic triggers (e.g., ischemia, exercise, hormones). For example, cardiac and skeletal muscle have robust angiogenic responses to exercise, in part because their elevated metabolic activity and hypertrophic growth trigger local VEGF release [44,45]. Meanwhile, the brain has a very limited response to exercise [46]. This reflects the importance of tissue metabolism and cell turnover in determining tissues’ responses to angiogenic cues.

On the other hand, arteriogenesis is best understood through its protective role following the occlusion of neighboring collaterals. As detailed by Korshunov [47], this is commonly observed in the coronary, middle cerebral, and femoral arteries during coronary artery disease, peripheral artery disease and stroke, respectively. However, arteriogenesis occurs even in the absence of vascular disease, ensuring that blood flow always meets the requirements of downstream organs [6,47]. For example, Hudlicka et al. [48] showed that exercise-induced arteriogenesis enhances perfusion and O_2_ delivery to the heart and skeletal muscle. Similarly, a study by Ferenczy et al. [49] indicated that progesterone influences arteriogenesis in uterine arteries during the menstrual cycle. Hence, exercise and hormonal influences appear to predict angiogenesis and arteriogenesis in health and disease. However, little is understood about the baseline expression of arteriogenic factors and their receptors (e.g., MCP-1, EGF) and how this determines the arteriogenic capacity of different collaterals.

Taken together, these observations suggest a feedback mechanism aimed at achieving optimal vascular diameter and density. In this model, periods of elevated metabolic demand or reduced O_2_ availability lead muscle cells into hypoxia, thereby triggering VEGF release. Most cell types produce VEGF under hypoxic conditions [50]. In the brain, astrocytes produce the most VEGF under baseline and anoxic conditions [51], but it remains unclear which other cell types might be especially responsive to hypoxia in other organs. VEGF acts as a potent angiogenic signal, causing endothelial cell proliferation, migration, and new blood vessel formation. Such neovascularization improves the blood supply to the area, enhancing O_2_ and nutrient delivery to meet tissue metabolic needs. As the tissue’s vascular network expands, oxygenation increases, potentially reducing further VEGF release and thus finely adjusting the vascular response to the muscle’s metabolic requirements [45]. This feedback loop is essential for the adaptation of tissues to varying physiological demands.

The cardiac SA node provides an interesting test to this model. The superior SA node carries a high density of micro vessels, whilst the inferior section of the node has a much lower density of micro vessels. The firing frequency of SA node myocyte in the superior section of the node is nearly 4-fold higher than in the inferior SA node [52]. Thus, it is intriguing to speculate that the angiogenic feedback model described above drives these regional differences in vascular density to match the activity of surrounding myocytes. Future experiments should investigate, for example, if VEGF, HIF, and/or TGFβ levels vary along the SA node.

### 2.4. What are the functional requirements for capillary growth?

As new capillaries form and reach their mature state, they must become capable of executing their key functions. Transportation is the canonical role of the vascular system, and this is understood to be the primary factor determining steady-state vessel density, i.e. cells must be within a sufficient distance of microvessels to exchange nutrients and waste. However, the modes of nutrient exchange vary greatly between tissues, as do the resting-state vessel densities and the kinetics and mechanisms of dynamically regulated blood flow. In this section, we will examine the roles of capillaries, and importantly, how different vascular cells fit together to define the anatomical and functional hallmarks of a mature capillary.

Respiring cells consume O_2_ and produce carbon dioxide, each of which diffuse through the endothelial layer by simple diffusion. Tight junctions prohibit most larger molecules from permeating between endothelial cells, whilst adherens junctions which provide limited transport of water and solutes [53]. Diffusion is the primary mode of trans-endothelial transport in the brain [53]. In contrast, the kidney glomerulus permits bulk fluid transport through fenestrated endothelial layers. In transcytosis, macromolecules are selectively transported across the endothelial layer via receptor-mediated endocytosis and pinocytosis [53]. Macromolecules are then encapsulated into vesicles and transported across the endothelial layer, diverted, or destroyed. This selectivity is particularly apparent in the blood-brain barrier (BBB), where the endothelium is less permeable to small molecules.

To fulfill its transport role, the vascular network must consistently deliver oxygenated blood to metabolically active tissues. Blood flow is largely controlled by pressure differences and vessel diameter. The diameter of arteries and arterioles is regulated by the contraction and relaxation of their SMCs. During the myogenic response, SMCs depolarize, activating L-type Ca_V_1.2 channels. Ca^2+^ entry through these channel increases intracellular [Ca^2+^] and activates cross-bridge cycling [54]. Membrane depolarization is opposed by the activation of voltage-gated K_V_1.5 and K_V_2.1 channels as well as large-conductance Ca^2+^-activated K^+^ (BK_Ca_) channels [55,56]. BK_Ca_ channels are activated by Ca^2+^ sparks produced by the opening of sarcoplasmic reticulum ryanodine receptors (RyR) [57]. Activation of K_V_ and BK_Ca_ channels oppose vasoconstriction by hyperpolarizing SMCs, which decreases the activity of Ca_V_1.2 channels. This decreases intracellular Ca^2+^, causing relaxation.

Endothelial cells regulate SMC contractility through two mechanisms. Activation of endothelial inward-rectifier K_IR_2.1 and small/intermediate-conductance Ca^2+^-activated K^+^ (S/IK_Ca_) channels hyperpolarizes the endothelial cell, transmitting the signal to neighboring SMCs through myo-endothelial gap junctions [58]. Vasoactive molecules (e.g., NO) are also produced, which diffuse through cell membranes to relax SMCs [59]. Both mechanisms are controlled by intracellular Ca^2+^ signaling via TRPV4 channels and inositol trisphosphate (IP_3_) receptors [3]. Endothelium-dependent vasodilation is activated by intralumenal shear stress [60], whilst SMC membrane stretch elicits vasoconstriction (myogenic tone) [61]. Combined, shear stress-induced vasodilation and myogenic tone underpin a versatile and reactive vascular network.

The vascular endothelium acts as a vital conduit for electrochemical messages. Endothelial cell-to-cell communication allows for capillaries to sense local signaling events and direct upstream arterioles to dilate and increase blood flow. This effect (functional hyperemia) is well-documented in the brain where neurons maintain exquisite spatiotemporal control of blood flow (neurovascular coupling). Two key mechanisms of endothelial cell-to-cell communication are known to facilitate capillary-to-arteriole communication: one propelled by extracellular $K^{+}\;\left(K_{o}^{+}\right)$ signals, and the other by ATP_o_ and ${Ca}_{i}^{2+}$ signals.

$K_{o}^{+}$-driven retrograde signaling occurs when K^+^ ions bind to the extracellular face of K_IR_2.1 channels, which become activated, conducting further K^+^ efflux and membrane hyperpolarization [2]. This propagates along the endothelial cell membrane, supported by gap junctions linking the cytosolic compartments of adjacent cells. The retrograde signal ultimately reaches upstream SMCs, which become hyperpolarized and relax.

A series of other channels support the capillary-to-endothelial signaling driven by endothelial K_IR_2.1. TRPA1 Ca^2+^ influx activates pannexin 1 (PANX1) channels, which bind to P2X receptors, triggering Ca^2+^ influx and thus NO production [62]. Reciprocal PANX1 and P2X activity propagates along the endothelium, summating with local Ca^2+^ signals from IP_3_R and TRPV4 channels to produce a range of signaling events which produce a graded vasodilation [3].

When does a newly-grown capillary develop these complex signaling abilities? How is mechanosensation translated into different vascular functions during sprouting angiogenesis and functional hyperemia? The transition between nascent and mature capillary function remain obscured.

To summarize, capillaries facilitate nutrient transport through divergent mechanisms matched to the requirements of their local tissues, and microvessel density is largely match to tissue requirements through the mechanisms of angiogenesis and rarefaction. Blood flow into microvascular beds is tightly regulated by SMCs and endothelial cells, which dynamically control the diameter of large arteries. However, microvascular beds also exert precise control over blood flow within and between capillaries. The anatomical and functional maturity of these capillaries is denoted by the presence of pericytes. Next, we will discuss the contribution of pericytes to capillary structure and function.

### 2.5. Pericytes are hallmarks of a mature capillary

Pericytes have emerged as central players in the autoregulation of local blood flow and as anatomical hallmarks of mature microvessels. Pericytes act as chaperones for angiogenic sprouting, controlling where sprouting may occur and therefore exerting their own regulatory influence over angiogenesis and long-term vascular development [32]. It is important to consider the transformative role played by pericytes as they guide nascent capillaries from early development into functional and anatomical maturity. To date, this is largely understood through the co-ordinate signaling of pericytes and endothelial cells via Tie2 and Angiopoietin-1, however much of the pericyte role in later stages of angiogenesis remains poorly defined.

In mature microvascular beds, most capillaries (~90%) are associated with pericytes [63], and their embedded position in the capillary basement membrane highlights their integral role in capillary function. Pericytes provide structural support to the membrane, limit transcytosis across neighboring endothelial cells, and enhance the expression and alignment of tight junction proteins [64]. These are all crucial features acquired in the final stages of capillary growth, however the processes underlying the final stage of capillary ‘maturation’ are unclear. Significant work is warranted to better understand how pericytes consolidate the endothelial layer, regulate permeability and enable regulated nutrient transport to local tissues.

Recent studies in the brain and retina have identified distinct pericyte sub-populations strategically positioned along the vascular branching pattern to fulfill distinct roles in maintaining vascular stability and function [65]. Ensheathing pericytes are found on pre-capillary arterioles and the proceeding 1st ~ 3rd order capillaries, where their robust, enveloping morphology provides substantial structural support [66]. As these capillaries branch further, mesh pericytes can be identified by their complex network-like structure and extensive branching [67], followed by thin-strand pericytes, which extend long, slender processes to provide discontinuous coverage of the capillary endothelium [67].

The contractile role played by pericyte subpopulations has been controversial. One study found that only 25–30% of brain pericytes contract and regulate blood flow – these are predominantly the mesh and ensheathing pericytes [68]. However, all pericyte subpopulations express the contractile apparatus required to constrict an associated capillary [69], and an eloquent study recently identified pericytes attached to 9th order cerebral capillaries which also regulate blood flow, simply with far slower kinetics than their upstream counterparts [34]. Hence, pericyte subpopulations control functional hyperemia to greatly varying degrees, endowing mature capillaries with diverse functionality [34,68]. Herein, we will refer broadly to ‘contractile pericytes’ to highlight the impacts of this diverse population upon capillary constriction and functional hyperemia, however it should be noted that different studies focus specifically on different pericyte subpopulations.

Contractile pericytes utilize similar excitation-contraction machinery to SMCs to contract long projections around the circumference of adjacent capillaries [65]. By tonically controlling capillary diameter, pericytes may respond to electrical and chemical signals from neighboring endothelial cells to dilate the capillary lumen. This positions them to exert dynamic and precise control over microvascular blood flow [65]. For example, pericytes link capillary dilation with local glucose metabolism through the actions of ATP-sensitive K^+^ (K_ATP_) channels, which modulate pericyte contractility to elevate blood flow towards highly metabolically active cells [70]. This form of metabolic signaling can also activate endothelial retrograde hyperpolarization, dilating upstream arteriolar smooth muscle to further elevate blood flow to active tissues [70]. Whilst this salient mechanism has recently been revealed in the brain, it is yet to be observed whether similar mechanisms control capillary blood flow in other organs, such as the heart [65]. Whilst local O_2_ and glucose consumption modulate local blood flow, the other mechanisms controlling local blood flow are an area of intensive investigation.

To summarize, pericytes are essential regulators of a diverse range of capillary functions, through the many stages of angiogenic growth, and also in mature capillary function [65]. However, their high surface area and intimate positioning on the vascular network may expose them to the effects of oxidative stress and elevated blood pressure, with highly deleterious consequences [65,71–73]. Next, we will discuss how microvascular dysfunction develops into the progressive loss of pericytes, capillaries, and functional tissues: microvascular rarefaction.

## 3. Microvascular rarefaction

In this section of the review, we turn our attention to microvascular rarefaction: the loss or reduction of small blood vessels (capillaries) within a tissue. This phenomenon can diminish blood supply to the affected areas, leading to a decrease in O_2_ and nutrient delivery and impaired removal of metabolic wastes. Microvascular rarefaction is observed in various pathological conditions, including hypertension, diabetes, and chronic heart failure, contributing to tissue ischemia and organ dysfunction [74–77].

**Fig. 4.**
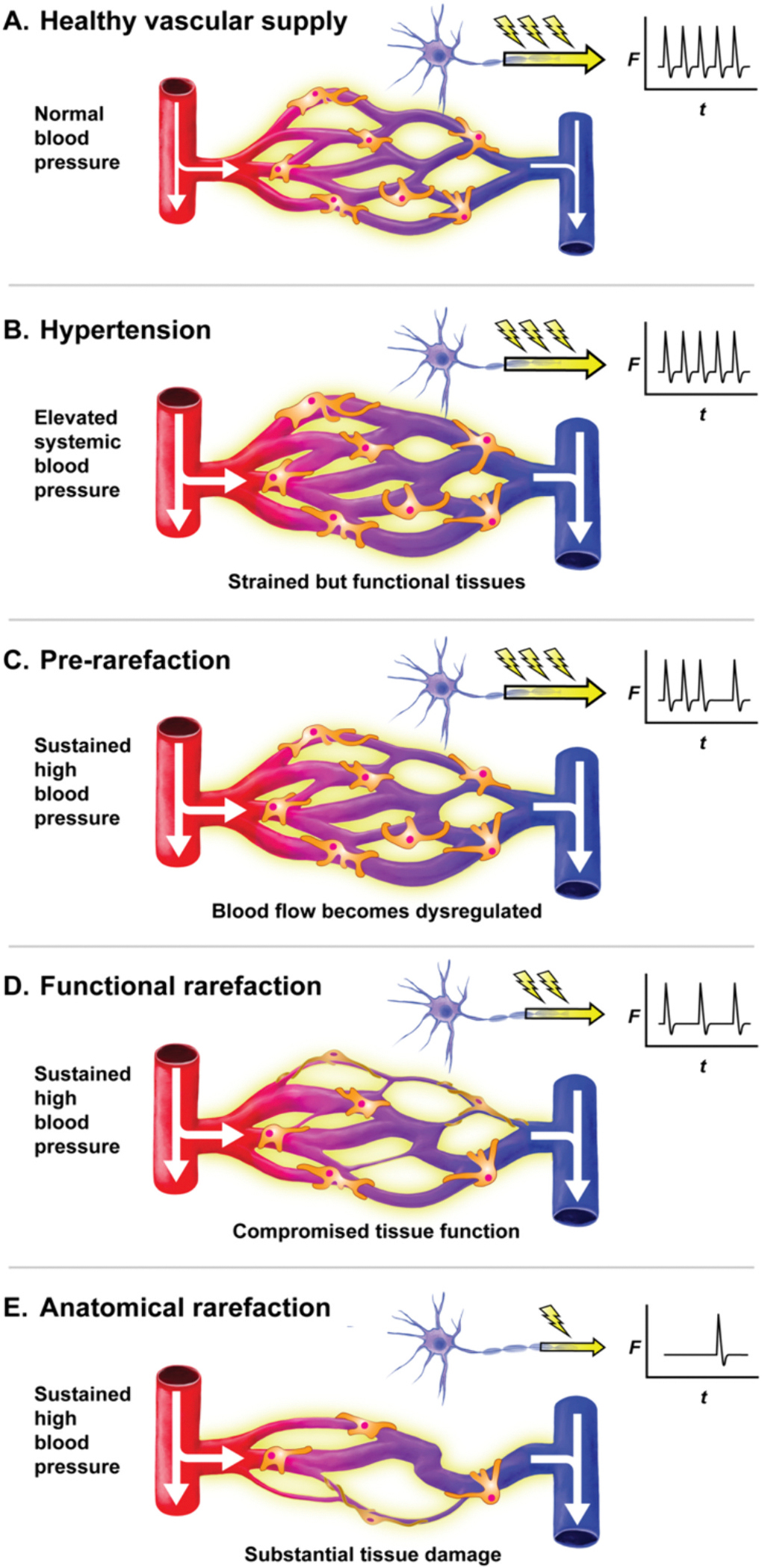
The functional consequences of vascular rarefaction in the brain. A healthy vascular supply (A) services metabolically active tissues and becomes strained during hypertension (B). Blood flow becomes dysregulated as vascular cells become damaged (C). Functional rarefaction occurs when capillaries no longer conduct blood flow (D). Tissues start to become dysfunctional as their metabolic requirements are no longer met. In anatomical rarefaction (E), capillaries begin to die and tissue function is severely blunted.

Briefly, we will now consider the different phases of vascular degeneration which lead to capillary death (Fig. 4). Diseases such as hypertension increase vascular resistance, causing capillaries to gradu ally sustain damage and accumulate toxic reactive oxygen species (ROS) (Fig. 4B) [77,78]. This causes a gradual decline in routine capillary functions. For example, blood flow can become dysregulated as electrical signaling and functional hyperemia are blunted. This phase of vascular dysfunction could be considered a “pre-rarefaction” period (Fig. 4C).

The major events of microvascular rarefaction are classified into two stages: functional and anatomical. Functional rarefaction occurs when blood vessels, most commonly capillaries, are no longer conductive to blood flow (Fig. 4D) [79]. Anatomical rarefaction occurs when endothelial cell death compromises entire capillaries, resulting in a loss of vascular density (Fig. 4E) [75,80]. As capillaries die, perfusion to tissues decreases leading to ischemia, hypoxia, and a buildup of metabolic by-products and ROS [75–77]. As a result, tissues become damaged and dysfunctional [75–77]. Notable examples of this progressive microvascular rarefaction progression include vascular dementia [81] and heart failure with preserved ejection fraction (HFpEF) [82].

### 3.1. Microvascular rarefaction in heart failure with preserved ejection fraction

HFpEF is clinical syndrome characterized by dyspnea, orthopnea, fatigue, edema, paroxysmal nocturnal dyspnea with preserved left ventricular ejection fraction ≤50% and impaired diastolic function [82]. Microvascular rarefaction is a proposed mechanism underlying the pathophysiology of HFpEF [74,82].

It is proposed comorbidities common to HFpEF such as obesity, diabetes, and renal failure create a systemic pro-inflammatory state which leads to microvascular endothelial inflammation by reducing endothelial NO bioavailability, cyclic guanosine monophosphate (cGMP) production, and protein kinase G activity by adjacent cardiomyocytes [74,83]. This leads to inflammatory cell infiltration and production of pro-fibrotic cytokines resulting in diastolic dysfunction due to altered cardiomyocyte function and extracellular matrix [74,84]. In addition, capillary endothelial inflammation increases the O_2_ demands of endothelial cells, resulting in hypoxia, hypercapnia, buildup of toxic oxidative species, and ultimately, endothelial cell dysfunction and death [83,85]. Subsequent loss of endothelial cells leads to loss of entire capillaries and decreased perfusion of cardiac interstitium during times of stress, resulting in concentric cardiac remodeling and decreased diastolic function [74,84].

Clinical studies of human heart failure have found that 6% of individuals with a heart failure-related admission have bradycardia and 1/6 had a bradyarrhythmia complicating their course [86]. Thus, indicating sinoatrial node dysfunction in HFpEF. While it is unknown if microvascular rarefaction leads to SA node dysfunction in HFpEF, we hypothesize that microvascular rarefaction occurs in the capillaries perfusing the SA node [87]. We propose that uncontrolled endothelial inflammation in SA node capillaries leads to endothelial dysfunction and cell death, ultimately leading to capillary retraction and anatomical rarefaction [87]. This causes decreased perfusion to the sinoatrial node. SA node cardiomyocytes subsequently suffer hypoxia, hypercapnia and decreased metabolic capabilities leading to SA cardiomyocyte dysfunction and eventual death [87,88]. Decreased SA node cardiomyocytes leads to SA node dysfunction and an inability to maintain stable HR resulting in the development of bradycardia and bradyarrhythmia in HFpEF [87,88].

Systemic pro-inflammatory states from comorbid conditions such as obesity, diabetes and hypertension provoke extensive endothelial inflammation and ROS generation [89]. This process can prevent angiogenesis, disrupting the negative feedback mechanism ensuring adequate O_2_ supply to all tissues [90]. Ultimately, microvascular rarefaction becomes a positive feedback mechanism whereby worsened vascular pathology drives tissue hypoxia and inflammation, blunting angiogenic processes and pushing the tissue towards a more hypoxic state [71,74,77].

In the cardiac circulation, several endothelial signaling pathologies have been identified which facilitate this shift [91]. For example, endothelial ROS are generated largely by nicotinamide adenine dinucleotide phosphate (NADPH) oxidases, which can become overexpressed or overactive in many disease states to produce huge amounts of endothelial ROS and subsequent cellular dysfunction [92]. In healthy tissues, this effect is buffered by the transcriptional factor Forkhead Box O3 (FOXO3), however FOXO3 functionality can also be lost in these disease pathologies [93]. One reason for this is that FOXO3 activity is dependent on NAD-dependent acetylases termed Sirtuins (SIRTs) [93]. The FOXO3 oxidative resistance pathway, and its cofactors SIRT1 and SIRT3 are critical protectors against endothelial ROS accumulation, particularly through the actions of SIRT3 in the mitochondria [91,94]. SIRT3 is notably deficient in the endothelial cells lining the coronary microvasculature during heart failure [90].

It is hypothesized that decreased perfusion to cardiomyocytes is a key factor in HFpEF disease progression [74,85]. Loss of endothelial SIRT3 expression allows ROS accumulation, which blunts NO production [91]. Decreased NO release reduces arteriolar vasodilation and perfusion to cardiomyocytes [91]. This phase of functional rarefaction worsens oxidative stress of cardiomyocytes and impairs diastolic function, ultimately leading to myocardial fibrosis [77].

These processes above represent the interruption of the angiogenic negative feedback system, and the establishment of a pathological positive-feedback system whereby endothelial dysfunction and functional rarefaction reduce tissue perfusion, causing hypoxia [74]. Where angiogenesis might normally resolve this in healthy tissue, oxidative stress prevents capillary growth and leads the vascular bed and surrounding tissue into a downward spiral [74,90].

### 3.2. Microvascular rarefaction in vascular dementia

The brain is a highly regulated organ requiring constant perfusion of both blood and cerebrospinal fluid within the limited space imposed by the skull [95]. This hindrance limits the capacity of cerebral feed arteries to dilate simultaneously [95]. Instead, parenchymal arterioles and capillaries must be selectively dilated to facilitate increased blood flow in extremely localized brain regions. Trains of action potentials and the associated neurotransmitter release consume huge amounts of ATP and even brief interruption to synaptic metabolism can severely impair neuronal function [96]. However, the brain does not carry a sufficient energetic reserve to satisfy its ATP requirements for more than a few minutes [97]. Given the high metabolic demands of neurons, substantial damage and loss of brain cells can occur during prolonged hypoxia [98]. Neurovascular coupling ensures that blood flow is directed towards highly active regions of the brain, satisfying neurons’ perfusion requirements within the limitations of the skull [99]. However, these intricate mechanisms become profoundly disrupted during vascular dementia [71,81].

Vascular dementia is a major disease originating from microvascular rarefaction. It is a progressive neurodegenerative disease accounting for 15–20% of all dementia cases worldwide [100]. There are several subtypes of vascular dementia including stroke induced dementia, multi-infarct dementia, small vessel disease, Cerebral Autosomal Dominant Arteriopathy with Subcortical Infarcts and Leukoencephalopathy (CADASIL), and mixed vascular-Alzheimer’s disease [101], which all lead to chronic hypoperfusion of brain tissue.

In vascular dementia, chronic hypoperfusion and thromboembolic events result in hypoxic brain parenchyma [81,100]. Instead of triggering satisfactory recovery of cerebral perfusion via angiogenesis, degenerative brain damage produces a positive feedback effect, where hypoxia drives tissue dysfunction, which exacerbates blood vessel death and hypoxia [81,101]. The proposed mechanisms for microvascular rarefaction in cerebral tissue occur through a combination of following mechanisms: impaired angiogenesis and active capillary regression; endothelial dysfunction and apoptosis; pericyte loss; and loss of intra-lumenal shear stress [77]. These factors are likely shared in other tissues at risk from microvascular rarefaction, such as the heart, eyes and skeletal muscle [74,75,80].

When the brain parenchyma becomes hypoxic in vascular dementia, pro- and anti-angiogenic factors expression shifts, tilting endothelial cells away from protective angiogenesis [71]. This impairs angiogenesis and induces vessel regression, leading to microvascular rarefaction, further worsening of tissue hypoxia [71].

These processes reflect those observed in other conditions where angiogenesis fails, and vascular beds degenerate into anatomical rarefaction. For example, oxidative stress is central to diabetes, hypertension, and stroke – three of the biggest risk factors for vascular dementia [81]. Indeed, pharmacological activators of SIRT3 appear to be neuroprotective in mouse models of vascular dementia [94], and a protective allele of the FOXO3 gene was recently shown to be protective against vascular dementia, much as it is protective against many other forms of cardiovascular disease [102].

In the early stages of vascular dementia, oxidative stress causes neurovascular blood flow to become dysregulated [71]. Neurovascular uncoupling occurs, whereby blood can no longer be directed towards neurons on-demand [71,81]. The pathologies underpinning this process are a subject of intense investigation, with endothelial cells and pericytes emerging as two essential mediators [1,12,62,65]. Briefly, endothelial cells lose the ability to communicate electrical signals and dilate the lumen of parenchymal arterioles and capillaries. As contractile pericytes are lost, capillaries with inadequate endothelial function lose all capacity to restrict and regulate their blood flow [71–73,103]. This has a knock-on effect to neighboring healthy vessels, as blood flow volume is directed into dysregulated capillaries [73]. This “flow steal” effect reduces the volume of blood flowing into the neighboring healthy capillaries, which experience reduced average flow velocity and sporadic blood flow stalling [73]. These flow defects fit the definition of “functional rarefaction” and may also underly part of the “cerebral hypoperfusion” measure diagnostic of vascular dementias.

Eventually, cerebral capillaries progress from functional rarefaction into anatomical rarefaction. Blood flow stasis, micro-embolisms and oxidative stress and hypoxia distort capillaries into tortuous structures [77]. When the brain parenchyma becomes hypoxic, pro- and anti-angiogenic factors expression shifts, tilting endothelial cells away from protective angiogenesis [71]. This impairs angiogenesis and induces vessel regression [71,77]. Endothelial cells progress from dysfunction into apoptosis, finally leading to capillary death [71,77]. Pockets of hypoxia form in the brain parenchyma where blood flow is absent [77]. This chronic hypoperfusion leads to neuronal loss and tissue death, progressing eventually into vascular dementia.

### 3.3. How does hypertension drive microvascular rarefaction?

**Fig. 5.**
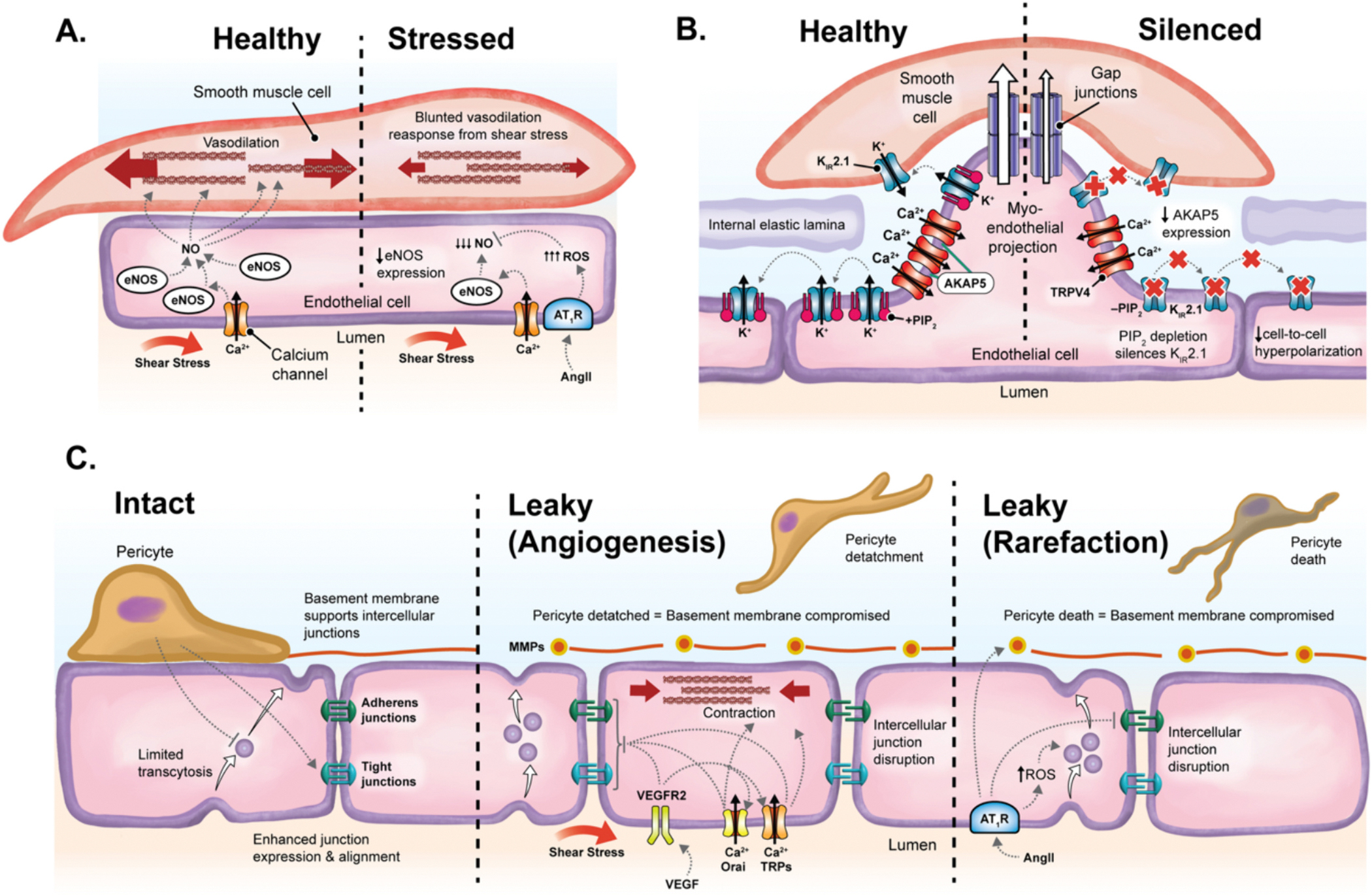
Endothelial dysfunction in vascular rarefaction. eNOS becomes downregulated, and increased ROS generation by AT_2_R disables NO-induced vasodilation (A). Silencing of K^+^ and TRPV4 channels compromises endothelial cell-to-cell communication and vasoreactivity (B). PIP_2_ depletion prohibits K_IR_2.1 channel hyperpolarization, and AKAP5 expression declines, dispersing and blunting the cooperative gating of TRPV4 channels. Finally, endothelial trans- and paracellular permeability is regulated by attached pericytes (C). This becomes disrupted during VEGF-induced angiogenesis and AngII-induced rarefaction, where pericytes detach and die, respectively.

Hypertension is a primary risk factor for capillary degeneration across various organ systems, as evidenced by the observed decrease in capillary density in both hypertensive rodents and humans, even before overt pathology manifests in major organs [80,104]. The link between early-stage hypertension and vascular rarefaction remains unclear, yet chronic high blood pressure promotes vasoconstriction, arterial hyper trophy, and fibrosis, thereby diminishing blood flow and directly damaging endothelial cells in both large arteries and capillaries. During hypertension, endothelial eNOS expression and function are reduced, leading to ‘uncoupling’ where eNOS inadequately produces NO and instead generates superoxide radicals. These radicals transform NO into peroxynitrite, promoting endothelial inflammation and DNA damage, and impairing vasodilation and hemodynamic responses (Fig. 5A) [91,105].

### 3.4. The endothelium undergoes electrical silencing during functional rarefaction

In the early stages of rarefaction, endothelial damage gives way to a loss of vasoresponsivity and failure to adequately control blood flow. This is attributed to the loss of endothelial hyperpolarization and ${Ca}_{i}^{2+}$ signaling capacity, which prohibit the endothelium from either transmitting vasoreactive signals, or translating them into local vasodilation through ${Ca}_{i}^{2+}$ signals (Fig. 5B).

One such effect originates with SK_Ca_ and IK_Ca_ channels, which decline functionally in a range of microvascular rarefaction pathologies [106,107]. These changes can originate from both transcriptional changes to channel expression [106] and also metabolic inhibition of membrane SK_Ca_ channels [107]. On the other hand, K_IR_2.1 channel functionality deteriorates when its cofactor, PIP_2_, becomes depleted [108]. This is notable in CADASIL, where PIP_2_ becomes depleted by disrupted epidermal growth factor receptor (EGFR) signaling [109]. As endothelial cells lose functional K^+^ channels, blood vessels lose the ability to transmit hemodynamic signals and blood flow become dysregulated [106,108].

During rarefaction, endothelial damage also gives rise to disrupted ${Ca}_{i}^{2+}$ signaling. One such example is the failure of AKAP5-mediated TRPV4 channel clustering at MEPs during hypertension [110]. In obese mice, oxidative species neutralize AKAP5 to prevent TRPV4 clustering [111]. This may underlie the loss of TRPV4 clustering in a range of pathologies, where cooperative gating, ${Ca}_{i}^{2+}$ signals and vasodilation all become compromised [111].

These detrimental processes together underlie a signaling blackout in the vascular endothelium during functional rarefaction. It is hypothesized that there may also exist a major shift in endothelial mechanosensation [60]. However, it remains unclear which of the many endothelial mechanosensors might become disrupted during rarefaction. Taken together, the molecular pathologies affecting endothelial cells during hypertension produce a profound loss in vasoreactivity.

### 3.5. Endothelial permeability is disrupted during angiogenesis and rarefaction

Vascular beds comprised of continuous capillaries feature an uninterrupted basement membrane and strong intercellular junctions which together regulate the movement of fluid and small molecules into surrounding tissues. In the brain, pericytes are shown to support inter-endothelial cell junction stability and limit vesicular transcytosis of large molecules across endothelial cells into the surrounding tissue [33]. However, these protective structures are disrupted during both angiogenesis and rarefaction (Fig. 5C).

In angiogenesis, intercellular junctions and basement membrane are disrupted, giving way to capillary growth. This originates largely from the actions of VEGF actions at endothelial VEGFR2, triggering TRP and Orai channel Ca^2+^ influx, intercellular junction disruption, MMP release and cell contraction [21,35]. Pericytes detach early in this process, disrupting intercellular junctions and removing their tonic suppression of trans-endothelial transport [32]. Combined, these effects mobilize endothelial cells whilst disrupting basement membrane and ECM in preparation for tip migration and stalk proliferation.

Similarities can be observed in AngII-induced hypertension, where AT_2_R-generated ROS upregulate transcytosis, disrupt inter-endothelial adherens junctions and release MMP to degrade the basement membrane [112]. The role of pericytes in rarefaction is still controversial: many labs have observed a fundamental role for pericytes in limiting permeability and regulating the BBB [33,64], however the Shih lab have demonstrated that mouse capillaries very rarely leak intravascular dyes within 21 days of selective pericyte ablation [73]. Further investigations are warranted to understand the circumstances surrounding pericyte death in different rarefaction pathologies.

### 3.6. Dysfunctional contractile cells set the scene for vascular dysfunction

The contribution of vascular SMCs to hypertension are profound and heavily documented [78]. Vascular SMCs undergo numerous pathologies which tilt them towards a hypercontractile phenotype. This constricts resistance arteries, elevating blood pressure and exerting strain on the downstream microvasculature, which becomes sensitized to microvascular rarefaction.

**Fig. 6.**
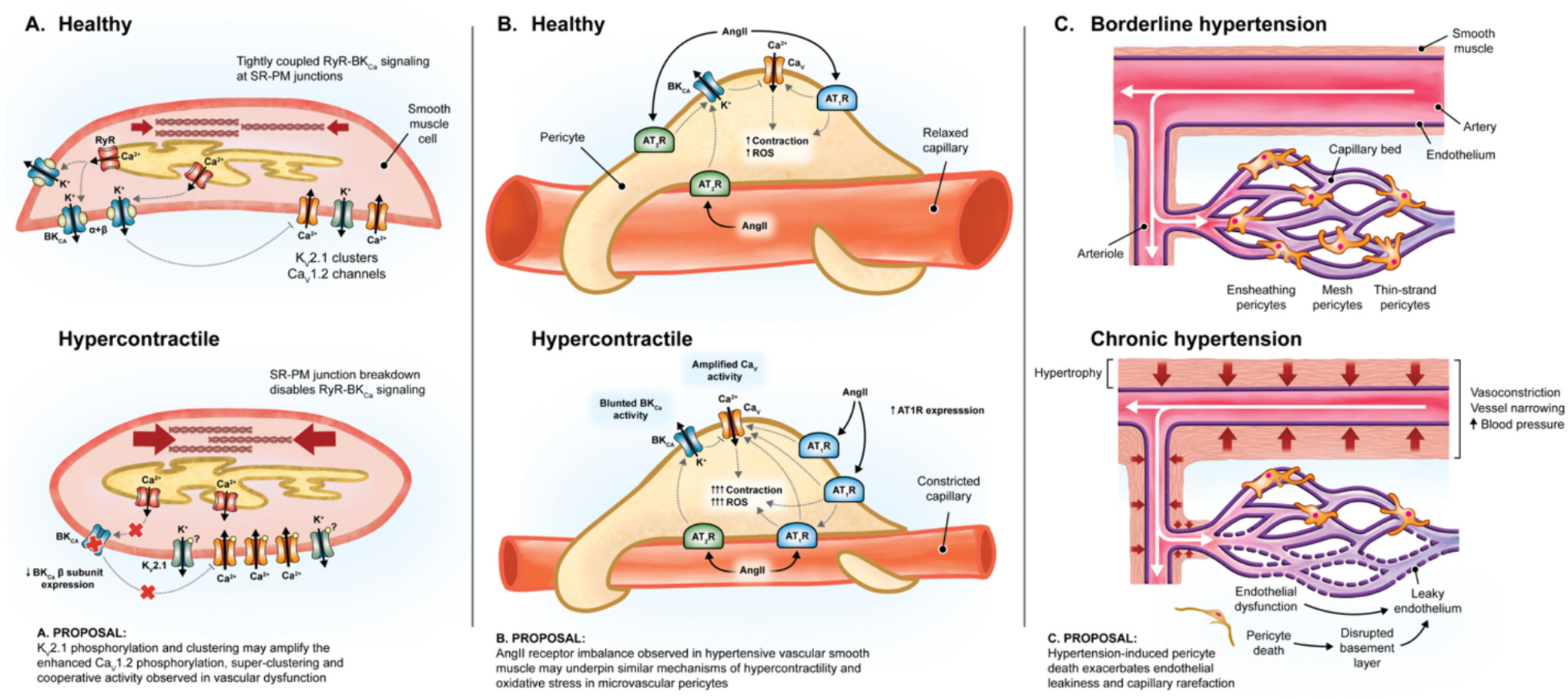
Proposed models of contractile cell dysfunction preceding rarefaction. During hypertension, SMCs become hypercontractile through several pathological processes (A). SR-PM domains break down, disrupting BK_Ca_-RyR coupling. Functional BK_Ca_ channels are depleted, whilst Ca_V_1.2 channels become hyperactive, increasing contractility. It is possible that K_V_2.1 channels may also contribute to Ca_V_1.2 super-clustering. In health, AngII-AT_1_R signaling drives contractility and oxidative stress in SMCs and pericytes (B). In hypertension, overexpressed AT_1_R increases SMC contractility and oxidative stress. This may be mirrored in pericytes and could initiate their loss in rarefaction. These proposed mechanisms could contribute to hypertrophic growth and microvascular pericyte death, which may exacerbate pre-existing endothelial dysfunction and permeability (C).

During hypertension, SMCs restructure at the micro-anatomical and molecular level (Fig. 6A). BK_Ca_ channel β1 subunit expression declines, reducing myocytes’ ability to repolarize and dilate resistance blood vessels [113]. Meanwhile, the SR becomes further distanced from the PM, physically uncoupling BK_Ca_ channels from RyR Ca^2+^ sparks [56]. Hence, BK_Ca_ channels become insufficient in their expression and responsiveness. Meanwhile, Ca_V_1.2 channels become hyperactive, pouring Ca^2+^ into the cytosol where it drives contractility. This occurs through an increased association with AKAP5, which anchors Ca_V_1.2 channel clusters alongside PKC and PKA, enabling PKC-driven cooperative gating Ca_V_1.2 channels [114]. Similarly, PKA has been found to phosphorylate the channel α1C subunit at S1928, super-clustering channels and driving cooperative gating during diabetes [54].

AngII is a key determinant of smooth muscle cell hypercontractility and oxidative stress. AT_1_R constitutively inhibits BK_Ca_ channels [115], and its signaling activates NOX, which generates harmful ROS (Fig. 6B). Simultaneously, AT_1_R-activated PKC upregulates Ca_V_1.2 activity and vascular contractility [114]. In healthy SMCs, this may be offset by the regenerative actions of AngII through AT_2_R, which activates BK_Ca_ K^+^ influx and vasorelaxation [116]. However, AT_1_R expression rises during hypertension, amplifying the SMC contractility and oxidative stress [117].

Human cerebral pericytes also express functional AT_1_R and AT_2_R, and like SMCs, exhibit contractile and ROS responses to circulating AngII (Fig. 6B) [118]. It remains poorly understood how pericytes behave prior to their death during hypertension and rarefaction, however it is tempting to speculate that a similar shift in AT_1_R/AT_2_R ratio might underlie the oxidative damage and functional rarefaction that precedes pericyte death during AngII-induced hypertension [119].

### 3.7. Structural changes to large arteries in hypertension may predict microvascular rarefaction

During hypertension, AngII-AT_1_R induces SMC hypertrophy which thickens the medial muscle of conductance and resistance arteries (Fig. 6C) [78]. This inward hypertrophic growth of large arteries reduces vessel responsiveness to changes in blood pressure as hypertrophic smooth muscle accumulates ECM components which develop into fibrotic tissue. Narrowed, unresponsive arteries are incapable of dilating appropriately to relieve elevated blood pressure and as a result, turbulent shear stress impacts downstream endothelial cells which generate ROS, which becomes amplified by AngII-AT_1_R signaling to drive microvascular dysfunction.

Pericytes are also lost during AngII-induced hypertension [119], however the progression of pre-hypertension into functional and anatomical rarefaction is poorly understood. Yet, there is an emerging consensus that pericyte death is an early event in rarefaction pathology [65,71]. We propose a model of AngII-induced hypertension where dysfunctional endothelial cells and pericytes produce ROS which leads to pericyte death (Fig. 6C). Pericyte death compromises the capillary basement membrane, exacerbating AngII-induced hyperpermeability across the endothelial layer [72]. As a result, nutrient delivery to the surrounding tissue becomes less efficient. These events, coupled with the loss of contractile pericyte-dependent control of microvascular blood flow [103], could underscore the progression of hypertension into functional rarefaction. From here, tissue and capillary function decline in tandem, as failure to revascularize the ailing tissue capitulates the transition into capillary death and anatomical rarefaction.

## 4. Conclusions and future studies

Here, we have discussed the biological mechanisms underpinning vascular growth and decline, with a special emphasis on capillaries and the mechanisms of sprouting angiogenesis and microvascular rarefaction. The mechanisms of endothelial cell proliferation and death have received great attention in these respective processes, as have the complex endothelial functions displayed in mature, functionalized capillaries. The interruption of endothelial signaling in rarefaction has been revealed recently. This and other mechanisms represent the gradual decay of capillary function in the buildup to anatomical rarefaction. It is interesting to consider how mature capillary functions, such as mechanotransduction shear stress-induced vasodilation and capillary-to-arteriole hyperpolarization, are developed in the vessel’s infancy. Furthermore, it remains to be assessed how these processes might operate in the surrounding vascular network during angiogenesis and arteriogenesis.

Studies show that angiogenesis and rarefaction rates profoundly affect disease progression. Excessive angiogenesis fuels cancer growth, while inadequacy impairs healing. Conversely, rarefaction causes ischemia in neurological and cardiovascular diseases. Future research should explore whether preventing capillary loss or restoring density and function can prevent or correct these conditions. The benefits of exercise-induced angiogenesis suggest such approaches could be fruitful.

Moreover, the emerging role of pericytes in health, angiogenesis and rarefaction presents a new paradigm of the dynamically-regulated capillary and particularly of the capillary’s guided growth and disruption during angiogenesis and rarefaction, respectively. More work is warranted to illuminate exactly why pericyte death predicts microvascular rarefaction. For example, do pericytes become hypercontractile during hypertension-induced rarefaction? Similarly, is ROS-induced pericyte death more likely to precede the permeability and death of its own attached capillary, or would this instead drive blood flow stealing and death of adjacent capillaries? These are questions to be assessed with in vivo models. Once understood, these factors might be used to predict and treat microvascular disease or serve as targets to modulate arteriogenesis and angiogenesis processes in health and disease.

## Data Availability

No data was used for the research described in the article.
